# Exploring the Potential of Drug Response Assays for Precision Medicine in Ovarian Cancer

**DOI:** 10.3390/ijms22010305

**Published:** 2020-12-30

**Authors:** Tanya Singh, Adam S. Neal, Neda A. Moatamed, Sanaz Memarzadeh

**Affiliations:** 1 Department of Obstetrics and Gynecology, David Geffen School of Medicine, University of California Los Angeles, Los Angeles, CA 90095, USA; tanyasingh@mednet.ucla.edu (T.S.); aneal@mednet.ucla.edu (A.S.N.); 2 UCLA Eli and Edythe Broad Center of Regenerative Medicine and Stem Cell Research, University of California Los Angeles, Los Angeles, CA 90095, USA; 3 Department of Pathology and Laboratory Medicine, David Geffen School of Medicine, University of California Los Angeles, Los Angeles, CA 90095, USA; nmoatamed@mednet.ucla.edu; 4 Molecular Biology Institute, University of California Los Angeles, Los Angeles, CA 90095, USA; 5 The VA Greater Los Angeles Healthcare System, Los Angeles, CA 90073, USA

**Keywords:** cancer therapeutics, precision medicine, drug response assays, tumor organoids, ovarian cancer, tumor spheroids

## Abstract

One of the major challenges in the treatment of cancer are differential responses of patients to existing standard of care anti-cancer drugs. These differential responses may, in part, be due to a diverse range of genomic, epigenomic, proteomic, and metabolic alterations among individuals suffering from the same type of cancer. Precision medicine is an emerging approach in cancer therapeutics that takes into account specific molecular alterations, environmental factors as well as lifestyle of individual patients. This approach allows clinicians and researchers to select or predict treatments that would most likely benefit the patient based on their individual tumor characteristics. One class of precision medicine tools are predictive, in vitro drug-response assays designed to test the sensitivity of patient tumor cells to existing or novel therapies. These assays have the potential to rapidly identify the most effective treatments for cancer patients and thus hold great promise in the field of precision medicine. In this review, we have highlighted several drug-response assays developed in ovarian cancer and discussed the current challenges and future prospects of these assays in the clinical management of this disease.

## 1. Introduction

Cancer is a major global health problem. According to the World Health Organization (WHO), cancer is the second leading cause of death behind cardiovascular disease, with one out of every six deaths being cancer-related. In the United States, the American Cancer Society estimates that in 2020 alone, there will be 1,806,590 new diagnoses and 606,520 cancer-related deaths [1].

Traditional modalities of cancer therapy such as surgery, radiation therapy, and chemotherapy still remain the standard of care for clinical management of most cancers [2]. Although these modalities have led to improvements in overall survival of many cancer patients, their impact can be somewhat limited to a subset of treatment sensitive tumors. In addition, these therapies can be associated with long term side-effects. Our growing understanding of the biology of cancer over the past few decades has shifted cancer treatment paradigms from organ-centric approaches to genotype-driven precision medicine approaches, leading to the development of more novel therapies for cancer: targeted therapies and immunotherapy [2,3]. While cancer immunotherapy is still in its early phase of development and is not yet widely directed as first-line treatment [4], targeted therapies have opened the door to personalized treatments for cancer patients focused on specific molecular alterations of individual tumors [5]. However, the successful implementation of targeted therapies requires development of assays or biomarkers that would allow clinicians to select and apply effective treatments.

Companion diagnostics that have the ability to identify patients who are most likely to benefit from a particular anti-cancer therapy are being increasingly integrated with drug-development processes and clinical trials [6]. HER2 overexpression by United States Food and Drug Administration (FDA)-approved assays was part of the approval for the use of trastuzumab (HERCEPTIN) in 1998 in selected patients with breast cancer [6]. The PD-L1 immunohistochemistry (IHC) 28-8 PharmDx assay is another FDA-approved companion diagnostic that assesses non-small cell lung cancer (NSCLC) patients to receive nivolumab (OPDIVO) based on overexpression of PD-L1 [6]. Most recently, in 2016, the FDA approved the VENTANA PD-L1 (SP142) assay as a companion diagnostic for selecting patients with NSCLC and bladder cancer for treatment with Atezolizumab (TECENTRIQ) [6]. In 2018, the FDA approved Olaparib (Lynparza) for the maintenance treatment of women with breast cancer susceptibility gene (BRCA)-mutated advanced epithelial ovarian, fallopian tube or peritoneal cancer with favorable response to first line platinum-based chemotherapy [7]. There are currently two FDA-approved companion diagnostics for Olaparib to identify patients with advanced ovarian cancer who harbor BRCA mutations: BRACAnalysis companion diagnostic (CDx) (Myriad Genetic Laboratories, Salt Lake City, UT, USA) and FoundationOne CDx (Foundation Medicine, Cambridge, MA, USA) [8]. Although biomarker-directed patient stratification has substantially improved clinical outcomes for cancer patients, its reach is limited to a small subset of targeted treatments. Moreover, response-predictive biomarkers are not available for most frontline anti-cancer therapies. Hence, alternative approaches are required that would allow the clinician to test the response of patient’s tumor cells to various available anti-cancer therapeutics.

Drug-response assays are in vitro platforms wherein live patient tumor cells are exposed to various chemotherapeutic and other agents in order to test their drug sensitivity. Development of such assays are specifically important in the context of ovarian cancer as most patients treated with the standard platinum-based chemotherapy eventually develop recurrent disease that is resistant to this treatment [9]. Due to the lack of effective second-line chemotherapies, the median overall survival of ovarian cancer patients with platinum resistant disease is about one year [9]. Hence, novel therapeutic strategies are an urgent need in the treatment of ovarian cancer patients. In recent years molecular targeted therapies have shown great promise for personalized treatment of ovarian cancer patients. For example, poly (ADP-ribose) polymerase (PARP) inhibitors and anti-angiogenic inhibitors are two FDA approved targeted drugs for ovarian cancer that have shown an encouraging progression free survival benefit [10]. However, these targeted drugs are only effective in a subset of patients. Hence, validated in vitro assays that can test individual tumor responses to chemotherapy or targeted therapies could be employed as a valuable clinical tool for individualized treatment of cancer patients.

The majority of drug-response assays involve culturing primary patient tumor cells in a 2D or 3D cell culture environment where they are exposed to cytotoxic drugs [11]. Performance of these assays may hinge on the type of cell culture technique being used. In this review, we have explored the potential of several 2D as well as 3D cell culture models as drug sensitivity screening tools in ovarian cancer. We have addressed the pitfalls and bottlenecks of these assays wherever possible. We believe that a better understanding of these preclinical models may help in promoting research initiatives focused on the development of assays for effective drug screening and clinical management of ovarian cancer.

## 2. Commercially Tested Drug-Response Assays in Ovarian Cancer

Chemo-response assays (CRAs) are ex vivo drug-response assays designed to characterize the sensitivity or resistance of a patient’s tumor cells to physician-selected, clinically applicable chemotherapy agents [12]. Most CRAs developed thus far share similar principles and procedures which include: (a) collection of primary patient tumor samples; (b) processing of tumor samples into single cells; (c) establishment of in vitro cell culture; (d) treatment with chemotherapeutic agents; (e) assessment of cell survival or death; and (f) statistical analysis and prediction of drug sensitivity [12].

Two commercially tested CRAs in the US include Microculture-Kinetic (MiCK) assay (DiaTech Oncology, Nashville, TN, USA) and ChemoFx assay (Helomics, Pittsburgh, PA, USA). Both assays utilize 2D cell culture systems. The MiCK assay is based on the principles of drug-induced apoptosis in which neoplastic cells are purified from patients’ tumor samples, seeded in a 2D cell culture environment and exposed to serially diluted chemotherapeutic drugs causing apoptosis in tumor cells [13]. The optical density (OD) of cells, a surrogate marker of apoptosis, is measured over time to create a density-by-time curve [13]. The extent of drug induced apoptosis is a measure of the tumor cells’ sensitivity to the tested drug. This assay has been applied to study the chemo-response of tumor cells of different types of cancers including hematologic [13], breast [14,15], lung [15], and gynecologic malignancies [15,16,17]. The MiCK assay has also been clinically tested in ovarian cancer patients. Some of these trials are outlined in Table 1.

The ChemoFx assay is another 2D culture based ex vivo assay designed to predict therapy sensitivity or resistance of a patient’s tumor cells to a variety of chemotherapy drugs [20]. After treating tumor cells with increasing doses of selected chemotherapeutic agents, the number of remaining live cells (cells without DAPI-stained nuclei) is quantified microscopically using automated cell-counting software. These data are used to generate dose-response curves and score tumors’ drug sensitivity as either “sensitive”, “intermediate sensitive”, or “resistant” [20]. In contrast to other CRAs, the ChemoFx assay has several advantages that may makes it clinically applicable [20]. First, it utilizes a cell-culture process that supports the growth of epithelial cells, decreasing the confounding effects of other cell types. This process is complimented by incorporating immunocytochemistry steps within its workflow to validate that the majority of cells are epithelial. Second, due to the low cellular volume required for this assay (as low as 35 mm^3^ of tissue), it is accessible for testing core needle biopsy samples. Finally, this assay is highly automated allowing use of a wide range of drug concentrations. This assay has been utilized to predict the chemo-response of several types of solid tumors, including ovarian cancer [18,19] and breast cancer [21]. Multiple clinical trials, both retrospective and prospective, have been reported on the clinical validity of this assay in ovarian cancer. Some of these trials are outlined in Table 1.

Beyond these two assays, there are several other 2D cell culture-based approaches that have been clinically evaluated in epithelial ovarian cancer as platforms for testing therapeutic responses. An overview of these assays is outlined in Table 2.

### Current Clinical Use of CRAs

According to the American Society of Clinical Oncology (ASCO) guidelines (2011) the use of chemotherapy sensitivity and resistance assays is not recommended outside of the clinical trial setting [26]. Most currently available CRAs utilize 2D cell culture models for testing drug sensitivity. Growing evidence demonstrates that cancer cells in 2D culture behave radically different than actual tumor cells in vivo [27]. Given limitations with existing 2D cell culture-based assays, many investigators have shifted their focus to utilization of 3D cell culture models which may be more representative of tumor architecture compared to 2D models.

## 3. Emergence of 3D Cell Culture Models for Drug-Testing in Ovarian Cancer

The importance of interaction between cells and an extracellular matrix (ECM) was pioneered by Mina Bissell in the early 1980s where she and her team postulated that a reciprocal and dynamic interaction between cells and their surrounding ECM can modulate gene expression [28]. Over time, investigations into this model led to the emergence of 3D cell culture techniques and 3D organoids became a preferred model for studying complex malignant tumors. In 3D culturing technique, cells are able to interact with each other and with the ECM to form organoids [29]. There are multiple lines of evidence that suggest the response of tumor cells to cytotoxic agents dramatically differs in 2D vs. 3D cell culture models [30,31,32]. Hence, these 3D culture models are being widely adopted in drug screening and drug toxicity assays.

Organoids, resembling mini organ structures, are 3D multicellular aggregates grown in an ECM and utilized to model human organ development and disease in a dish [29,33]. Organoids can be derived from tissues, primary tumors, cancer cell lines, and normal stem cells including embryonic stem cells and induced pluripotent stem cells when embedded within ECM hydrogels [29,33,34,35]. Due to their intrinsic ability of self-organization, they are thought to retain the identity of their tissue of origin [29,33]. There is some evidence supporting that genomic alterations are recapitulated in matrix-dependent organoid cultures [36,37]. As reviewed by Tibbit and Anseth [38], ECM hydrogels play an important role in the generation of organoids as they act as a scaffold for cell growth, promote cell adhesion, and enable proper transport of nutrients, gases, and growth factors to cells (Table 3).

Similar to organoids, spheroids are compact 3D multicellular structures. Although these terms are often used interchangeably, they represent two distinct models of 3D culture. Spheroids are cultured under non-adherent conditions in serum-free media [39] and may be generated from tumor tissue [40] or cancer cell lines [41]. Compared to 2D cell culture, spheroids may more closely resemble tumors due to their 3D structure [42]. In addition, spheroids are thought to be enriched for cancer stem cells (CSCs) [39,43]. Spheroid cultures may be relevant in ovarian cancer as such cell aggregates are naturally found in ascites, free-floating tumor cells found in the abdomen of patients, an important mode of distant metastasis [44,45]. In experimental models, ovarian cancer spheroids may contribute to disease progression, metastasis, and chemotherapy resistance [46,47]. Spheroid cultures have been used to investigate conditions in which CSCs can be grown, and also in evaluating the efficacy of therapies targeting ovarian cancer. Some of the applications of spheroid culture in ovarian cancer are outlined in Table 4.

Similar to spheroids, organoid 3D cultures can be utilized as tools for drug sensitivity assays because they also better recapitulate cell-cell interaction in a compact, self-organized structure compared to 2D cultures. Each model (spheroids vs. organoids) has its own advantages and disadvantages as reviewed by Gilazieva et al. [64]. For example, spheroid models are thought to be enriched for a cell population with a CSC phenotype; however, these models may not fully recapitulate the tumor architecture [64]. On the other hand, organoid models are more architecturally and functionally similar to their parental tumors and can be maintained in culture long term through passaging [64]. For use in drug testing assays, particular consideration should be given to the limitations associated with each model. For example, due to their compact 3D structure tumor spheroids may create a drug penetration gradient, while drug responses of organoids may be impacted by the ECM in which cells are embedded [64].

### Applications of Organoid Culture Models in Ovarian Cancer

**Figure 1:**
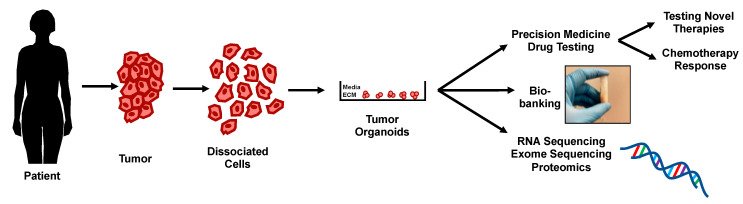
Overview of potential applications of tumor-derived organoids in ovarian cancer.

Tumor-derived organoids are patient-specific cancer organoids that are capable of partially preserving different tumor cell types as seen in vivo [65]. These organoids are generated from tumors obtained after surgical excision, biopsy, ascites, or pleural effusion samples. Primary tumor-derived organoids offer several advantages as preclinical models for drug testing: they are less resource intensive, can be readily expanded long term compared to patient-derived xenografts (PDXs) and are compatible with high throughput drug screening [66,67]. Tumor-derived organoids faithfully retain some of the biological properties of their parental tumors, even in long-term ex vivo expansion, making them a potentially better model compared to traditional cancer cell lines [36,68]. Hence, tumor-derived organoid models can help fill the gaps in cancer treatment research by complementing cell line and xenograft-based drug studies (Figure 1).

To fully utilize the potential of primary tumor-derived organoids as a platform for personalized medicine in cancer patients, scientists and drug companies are exploring the potential of large biobanks of organoids that may be used in predicting treatment response. Biobanks of patient-derived tumor organoids have been established for many tumors including colorectal [69], prostate [70], and breast cancer [71]. In these studies, patient-derived organoids were established to capture the heterogeneity and genetic landscape of their parental tumors and were further utilized as a platform to investigate therapeutic drug responses.

One short-term patient-derived tumor organoid study of high grade serous ovarian cancer was reported by Hill and colleagues wherein their research group established 33 organoid lines from 22 ovarian cancer patients [72]. These organoids were tested for defects in homologous recombination and replication fork protection and were used as a platform to predict therapeutic responses to PARP inhibitors [72]. Kopper et al. utilized tumor samples from 32 patients to establish 56 organoid lines, including the main subtypes of ovarian cancer [36]. They optimized protocols allowing these organoid lines to be genetically manipulated and transplanted in mice. This study also provided a proof of concept that organoid-derived xenografts can be used for testing anti-cancer drug sensitivity [36]. Maenhoudt and colleagues developed yet another ovarian cancer biobank derived from predominantly high grade serous ovarian cancer patients as preclinical models for drug screening [73]. Hoffmann et al. established 15 organoid lines from HGSOC tumor specimens that recapitulated the mutational profile and phenotype of their original tumors [74]. Using organoid cell culture models, several investigators have evaluated the therapeutic response of ovarian cancer cells to various chemotherapeutic agents. Some of these studies published in the past decade are outlined in Table 5.

**Figure 2:**
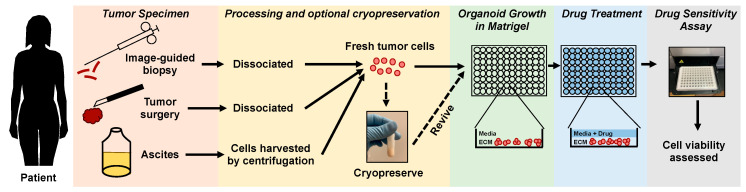
A workflow for testing drug response of ovarian cancer organoids using a high-throughput drug assay.

With vast experience in ovarian cancer research, we have collaborated with colleagues and contributed to the development of a 3D organoid based platform for high throughput drug screening [77]. In this assay, tumors retrieved from patients through institutional review board (IRB)-approved protocols are mechanically and enzymatically dissociated. These cells can either be cryopreserved or used freshly in the assay. Dissociated tumor cells are suspended in Matrigel and plated around the rim of a well in a 96-well plate to form a miniring, allowing the growth and formation of multiple organoids within the same well [77]. In this assay, tumor organoids are allowed to grow for two days followed by treatment with drugs in a dose-dependent manner for three days [77]. Following treatment, organoids are released from the Matrigel using dispase and cell viability is assessed by an ATP-based cell viability assay [77]. A workflow for assessing drug response in this assay using samples obtained from ovarian cancer patients through image-guided biopsies, surgical specimens, and ascites is shown in Figure 2.

**Figure 3:**
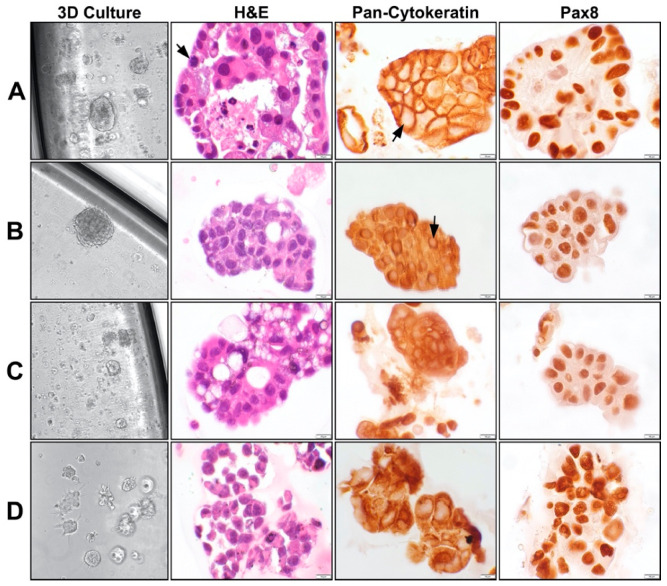
Morphology of platinum-resistant organoids in culture and their respective histology. (**A**–**C**) Organoids from three independent patients with platinum resistant high-grade serous carcinomas. (**D**) Organoids derived from platinum resistant ovarian cancer cell line OVCAR8. All four organoid models were established in Matrigel-based adherent 3D cell culture. Hematoxylin and eosin (H&E) staining show clusters of malignant cells with pleomorphic nuclei, mitoses (arrow), and necrosis (in A panel). Pan-cytokeratin staining show positivity in the narrow rims of the cytoplasm surrounding the pale staining nuclei (arrow), confirming epithelial origin of the cells. Pax8 shows nuclear staining of the malignant cells, consistent with Mullerian origin of the tumors. The morphological features and the immunohistochemical reactions are remarkably similar in all organoid cultures. All 3D culture organoid pictures are taken with a 20 × objective. All microscopic pictures are taken with a 60 × objective.

As a drug testing platform, this approach offers several advantages [77]. First, this assay is clinically accessible as it allows formation of organoids from a variety of tumor samples including solid tumors, ascites, and pleural effusion. Second, due to the low cellular input needed, biopsy samples can also be tested using this assay. Third, assay results are obtained within a week from collection of the clinical sample making it compatible with the timeline for therapeutic decision making [77]. The histology of organoids can also be studied when plated in larger culture dish wells. [82]. Examples of such organoids derived from patients diagnosed with platinum-resistant high-grade ovarian cancer and from a platinum-resistant ovarian cancer cell line are shown in Figure 3.

## 4. Advanced 3D Cell Culture Models as Preclinical Drug Screening Platforms

**Figure 4:**
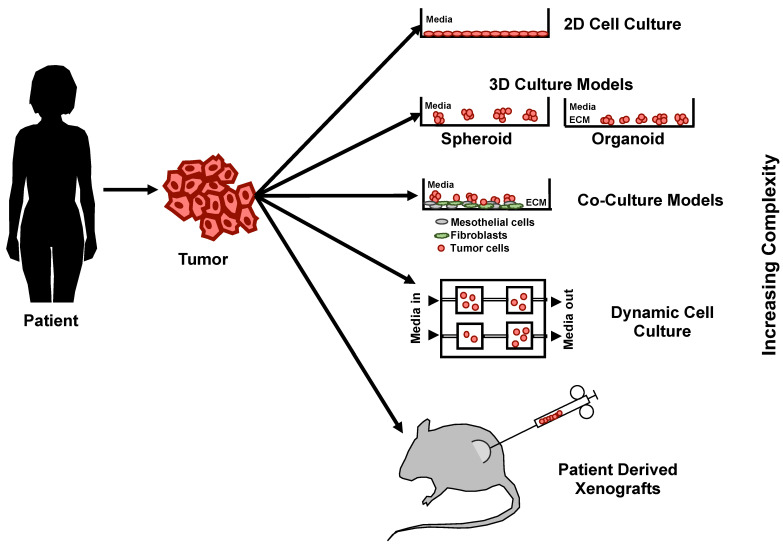
Evolution of pre-clinical models in ovarian cancer for drug testing.

The tumor microenvironment (TME) plays a very important role in disease progression and metastasis [83]. Ovarian cancer cells are known to metastasize to the peritoneal cavity and spread to the omentum [84]. During this process, cancer cells typically interact with non-transformed cells like mesothelial cells and omental fibroblasts. Hence, to design or screen new therapeutics targeting ovarian cancer cells, it is imperative that drug testing models mimic the TME. Significant progress has been made in the development of 3D culture models to replicate the metastatic niche of ovarian cancer cells (Figure 4).

Co-culture based models are advanced cell culture techniques where tumor cells are grown together with cells such as fibroblasts or stromal cells that support their growth and development [85]. Co-cultures may be of two types: direct and indirect [86]. In the direct type, different cell types are mixed prior to plating and cultured together; whereas, in the indirect type, two cells types are separated by a physical barrier. As reviewed by Hoarau-Véchot et al., co-cultures are feasible using either 2D or 3D models [87]. However, co-cultures may also present challenges in downstream analyses due to the presence of more than one cell type [87]. Some of the notable co-culture 3D models established in ovarian cancer are outlined in Table 6.

Dynamic 3D models are another clinically relevant cell culture system capable of simulating the ovarian cancer TME. Some of these models utilize microfluidics to continuously feed cultured cells with fresh media and remove toxic waste products [98]. Compared to 3D static cultures, this technique may be more representative for drug screening as it can simulate circulation rates and perfusion observed in human tissues. Microfluidic set ups can be used for long term tumor organoid/spheroid cultures and are generally coupled with miniaturized features that require a smaller number of cells and reduced amount of drugs to perform the assays [98,99]. Some of the key dynamic 3D cell culture platforms developed in ovarian cancer are outlined in Table 6.

## 5. Concluding Remarks and Future Directions

One of the biggest challenges in cancer drug development is the selection of appropriate models for preclinical research. A preclinical model should ideally recapitulate the heterogeneity and genetic landscape of the tumor as closely as possible and should also be compatible with high throughput drug screening. The three main preclinical models for drug testing include cancer cells grown in 2D culture, organoids/spheroids grown in 3D culture, and in vivo patient-derived xenograft models. 3D cell culture models may emerge as the most promising tool in preclinical research as they combine two strengths: (i) the ability to recapitulate tumor architecture, and (ii) compatibility with timely high throughput drug screening.

In this review we have highlighted the significance of chemotherapy-response assays in ovarian cancer. However, no ideal model (either 2D or 3D cell culture based) has been established or FDA-approved for clinical use yet. There are inherent challenges associated with developing these assays including (a) intra-tumoral heterogeneity, (b) inter-tumor heterogeneity between primary and metastatic sites from the same patient, and (c) absence of a tumor microenvironment. Intra-tumoral heterogeneity is known to play an important role in metastasis, invasion, therapy-resistance and tumor relapse [100]. The existence of multiple cell populations with differential therapeutic drug responses within the same tumor site may decrease the predictive accuracy of CRAs. Similarly, inter-tumor heterogeneity may also lead to differential therapeutic responses especially in diseases such as ovarian cancer where tumor recurrences tend to be metastatic and therapy resistant. The predictive value of these assays may be further limited by the absence of a tumor microenvironment. A vast source of existing literature suggests that other tumor components, such as fibroblasts, endothelial cells, ECM components, and infiltrating immune cells are important in disease progression, and hence should potentially be incorporated into drug response assays to make more accurate predictions on the chemosensitivity profile of tumor cells. In this context, the emergence of co-culture methods and dynamic 3D cell culture approaches hold great promise as pre-clinical tools for disease modeling, drug screening, and cytotoxicity testing.

In summary, this article examines existing chemo-response assays and their potential in improving the clinical outcome of ovarian cancer patients by predicting therapeutic responses ex vivo. To be adopted for widespread clinical use, these assays should employ cell culture models that recapitulate actual tumor architecture, incorporate supporting tumor microenvironment cells for better modeling of disease in vitro, and be compatible with automation and high throughput analysis in a cost-effective manner. For clinical translation, it is also important that these assays are validated through well-designed prospective and blinded multi-center clinical trials. We believe that a reliable bioassay-directed selection of treatment not only could improve patient quality of life but also reduce the economic burden due to costs associated with administration of less effective treatment regimens. Hence, we hope that continued optimization of these assays may help provide an effective precision medicine approach for individualized treatment of cancer patients.

## Figures and Tables

**Table 1:** Clinical trials with commercially available kits in ovarian cancer.

MiCK Assay				
Study	Study Type	Patient Population	Results ^1^	Reference
Salom et al. (2012)	Prospective	*n* = 104; epithelial ovarian cancer patients	OS of chemonaive patients with stage III or IV disease was longer when treated with assay-predicted chemotherapy	[17]
Bosserman et al. (2012)	Prospective and non-blinded	*n* = 44; breast cancer, non-small cell lung cancer, ovarian cancer, and others (*n* = 2 ovarian cancers)	Patients receiving assay-predicted chemotherapy demonstrated improved median OS compared to patients who were treated empirically	[15]
**ChemoFX Assay**				
**Study**	**Study Type**	**Patient Population**	**Results ^1^**	**Reference**
Herzog et al. (2010)	Retrospective	*n* = 192; ovarian cancer patients	A trend towards increased OS was seen in patients who received a treatment also found efficacious in vitro	[18]
Rutherford et al. (2013)	Prospective	*n* = 262; recurrent or persistent ovarian cancer	Improved PFS and OS for patients with assay-sensitive tumors compared to resistant or intermediate response based on in vitro assay.	[19]

^1^ PFS: Progression free survival, OS: Overall survival.

**Table 2:** Overview of 2D cell culture-based drug-response assays in ovarian cancer.

Study	Study Type	Patient Population	Cell Viability Assessment	Results ^1^	Reference
Konecny et al. (2000)	Non-randomized	*n* = 38; FIGO stage III ovarian cancer patients	ATP-based assay	Patients predicted to be sensitive showed a trend for increased PFS and OS compared to assay-predicted resistant patients	[22]
Sharma et al. (2003)	Prospective and non-randomized	*n* = 44; chemotherapy-treated recurrent epithelial ovarian cancer patients	ATP-based assay	Patients were treated according to the assay-predicted results. The overall response rate was 61% for evaluable patients	[23]
Loizzi et al. (2003)	Retrospective and non-randomized	*n* = 100; recurrent ovarian cancer	Tritiated thymidine uptake drug resistance assay	For patients with platinum-sensitive disease, 1-year OS and PFS were increased when treated with assay-directed chemotherapy compared to the control group.	[24]
Cree et al. (2007)	Prospective, randomized	*n* = 180; platinum-resistant recurrent ovarian cancer patients	ATP-based assay	Patients treated with assay-predicted chemotherapy regimens demonstrated a small trend toward improved PFS and response rate compared to physician’s choice treated patients (not statistically significant).	[25]

^1^ PFS: Progression free survival, OS: Overall survival.

**Table 3:** Types of extracellular matrix (ECM) gels used in 3D organoid culture.

Natural ECM Gels	Synthetic ECM Gels
Formed from extracellular matrix components or materials derived from biological sources such as chitosan and alginate.	Made of non-natural molecules
Biocompatible and bioactive	Inert molecules
Complex composition with presence of undefined endogenous components	Defined components
Altering properties of these gels can be difficult	Easily manufactured in a cost-effective manner, highly reproducible
Restricted clinical applications due to presence unknown endogenous factors	Used for clinical applications as well as fundamental studies of cell physiology
**Examples:** alginate, collagen, Matrigel, fibrin, hyaluronic acid	**Examples:** Poly(2-hydroxy ethyl methacrylate), Polyethylene glycol, Poly(vinyl alcohol)

Table summarized from Tibbitt and Anseth (2009), [38].

**Table 4:** Applications of spheroid culture models in ovarian cancer.

Study	Outcomes of the Study	Reference
Xing et al. (2005) Xing et al. (2007)	Analysis of spheroid culture from ovarian cancer cell lines revealed higher expression of p27 protein in spheroids associated with drug resistance to taxol	[48,49]
Kryczek et al.(2012) Silva et al. (2011)	CD133 and ALDH are identified as CSC markers of ovarian cancer using spheroid culture models	[50,51]
Xu et al. (2014)	E-cadherin plays an important role in spheroid formation and drug resistance to cisplatin.	[52]
Ishiguro et al. (2016)	ROCK inhibition of ovarian cancer spheroids may promote CSC phenotype	[53]
Raghavan et al. (2015)	Developed a novel 384-well hanging drop tumor spheroid platform used to test sensitivity to cisplatin chemotherapy.	[54]
Aihara et al. (2016)	Developed a novel 3D cell culture technique using FP001 polymer for screening of anticancer agents. FP001 facilitated homogenous spheroid culture.	[55]
Raghavan et al. (2017)	Developed a patient-derived 3D hanging drop spheroid platform with ALDH+ CD133+ ovarian cancer cells to screen the effects of chemotherapy drugs	[56]
Chen et al. (2017) Lu et al. (2019)	Activation of STAT3 plays an important role in formation of epithelial ovarian cancer spheroids and regulating putative stem-like cell markers	[57,58]
Yang et al. (2019)	Role of bcl-2 was explored in ovarian cancer spheroids in response to platinum-drugs	[59]
Rashidi et al. (2019)	Developed an in vitro 3D model to study stemness and chemoresistance in ovarian cancer. Serial passaging of spheroids using this technique demonstrated enrichment of cells with stem cell markers and emergence of platinum-resistance phenotype.	[60]
Shuford et al. (2019)	Developed an ex vivo patient derived 3D spheroid model for drug testing. A correlation between clinical response to therapy and in vitro response was seen in some patients.	[61]
Boylan et al.(2016) Boylan et al. (2020)	Cell adhesion molecule Nectin-4 may be involved in ovarian cancer spheroid formation	[62,63]

**Table 5:** Overview of organoid-based drug response assays in ovarian cancer.

Study	Type of ECM	Results	Reference
Loessner et al. (2010)	Bioengineered PEG-based hydrogel matrix	Developed a 3D cell culture platform using a biomimetic synthetic hydrogel. The resulting multicellular structures were tested for sensitivity to paclitaxel compared to 2D monolayer culture.	[75]
Yang and Zhao (2011)	Nanofiber scaffold based 3D cell culture	Ovarian cancer cells grown in 3D on nanofiber scaffold were found to exhibit higher therapeutic resistance to anti-cancer drugs, like 5-FU, paclitaxel, and curcumin compared to conventional 2D cell culture.	[76]
Hill et al. (2018)	Matrigel-based	Developed short-term patient-derived ovarian cancer organoids for drug screening analyses.	[72]
Phan et al. (2019)	Matrigel-based	Developed a high throughput drug screening platform to evaluate the therapeutic response of tumor organoids derived from clinical samples and cell lines.	[77]
Kopper et al. (2019)	Basement membrane extract-based	Developed patient-derived organoid lines from main subtypes of ovarian cancer as a platform for drug screening assays.	[36]
Maenhoudt et al. (2020)	Matrigel-based	Developed patient-derived organoid lines from predominately high grade serous ovarian cancers and performed mutational analyses and chemosensitivity assays.	[73]
Chen et al. (2020)	Basement membrane extract-based	Developed a short duration organoid culture platform derived from HGSOC malignant effusion specimens and utilized it for drug sensitivity testing	[78]
de Witte et al. (2020)	Basement membrane extract-based	Utilized a patient-derived organoid (PDO) platform to assess the chemotherapy response to various drugs. The in vitro drug response of 7 PDOs treated with carboplatin and paclitaxel correlated with clinical outcomes seen in those patients.	[79]
Nanki et al. (2020)	Matrigel-based	Developed a PDO platform that recapitulated the in vivo architecture and genetic signature of original ovarian cancer tumor. This was utilized for drug sensitivity testing using 23 FDA-approved drugs	[80]
Zhang et al. (2020)	Matrigel-based	Modelled HGSOC by genetically manipulating mouse fallopian tube epithelium. Sensitivity of derived organoids was tested in drug assays.	[81]

**Table 6:** Advanced 3D cell culture models developed in ovarian cancer.

Co-Culture 3D Models		
Study	Purpose of Model	Reference
Kenny et al. (2007)	Developed a 3D co-culture model to study ovarian cancer metastasis to omentum. The model demonstrated that attachment and invasion of ovarian cancer cells is promoted by fibroblasts but may be inhibited by mesothelial cells.	[88]
Kenny et al. (2015)	Developed a high throughput drug screening assay using primary human 3D co-culture incorporating fibroblasts, mesothelial cells, and ovarian cancer cells.	[89]
Wan et al. (2017)	Developed a 3D co-culture model using human endothelial cells and ovarian cancer cells to mimic the interaction between the two types of cells in cancer. Utilized this model for anti-cancer drug testing using paclitaxel and cisplatin.	[90]
Long et al. (2018)	Developed a 3D co-culture platform using tumor-associated macrophages (TAMs) and cancer cells to form ovarian cancer organoids.	[91]
Loessner et al. (2019)	Developed a 3D co-culture model using ovarian cancer and mesothelial cells. The model demonstrated a potential increase in cancer cell proliferation in vitro and in vivo compared to monoculture.	[92]
Hedegaard et al. (2020)	Developed a 3D co-culture model with human umbilical vein endothelial, human mesenchymal, and ovarian cancer cells using a novel peptide-derived hydrogel as alternative to Matrigel	[93]
**Dynamic 3D Cell Culture Models**		
**Study**	**Purpose of Model**	**Reference**
Li et al. (2017)	Developed a dynamic 3D co-culture system that mimics the interaction between ovarian cancer cells and mesothelium in a microfluidic device.	[94]
Arellano et al. (2017)	Utilized a high-throughput, microfluidic-based platform for in vitro drug testing of ovarian cancer cells	[95]
Masiello et al. (2018)	Developed a novel dynamic cell culture platform using an orbital shaker for generating ovarian cancer spheroids by mimicking in vivo dynamic tumor microenvironment using fluid shear stress.	[96]
Flont et al. (2020)	Developed a microfluidic based co-culture model with fibroblasts and ovarian cancer cells. This platform was utilized to evaluate the potential cytotoxic effects of photosensitisers.	[97]

## Abbreviations

2D	Two dimensional
3D	Three dimensional
ASCO	American Society of Clinical Oncology
BRCA	Breast cancer susceptibility gene
CDx	Companion diagnostic
CRA	Chemo-response assay
CSCs	Cancer Stem Cells
DAPI	4′,6-diamidino-2-phenylindole
ECM	Extracellular matrix
FDA	United States Food and Drug Administration
H&E	Hematoxylin and eosin
HER2	HER2/neu, receptor tyrosine kinase erbB-2
IHC	Immunohistochemistry
IRB	Institutional review board
mm^3^	Cubic millimeters
NSCLC	Non-small cell lung cancer
OD	Optical Density
OS	Overall survival
PARP	Poly (ADP-ribose) polymerase
Pax8	Paired box gene 8
PD-L1	Programmed death ligand 1
PDX	Patient derived xenograft
PFS	Progression free survival
TAM	Tumor-associated macrophage
TME	Tumor microenvironment
US	United States
WHO	World Health Organization
